# Matchout deuterium labelling of proteins for small-angle neutron scattering studies using prokaryotic and eukaryotic expression systems and high cell-density cultures 

**DOI:** 10.1007/s00249-016-1186-2

**Published:** 2016-11-14

**Authors:** O. Dunne, M. Weidenhaupt, P. Callow, A. Martel, M. Moulin, S. J. Perkins, M. Haertlein, V. T. Forsyth

**Affiliations:** 1 Department of Structural and Molecular Biology, University College London, Gower Street, London, WC1E 6BT UK; 2 Institut Laue Langevin, 71 avenue des Martyrs, 38042 Grenoble Cedex 9, France; 3 University Grenoble Alpes, CNRS, LMGP, F-38000 Grenoble, France; 4 Macromolecular Structure Group, Faculty of Natural Sciences, Keele University, Staffordshire, ST5 5BG UK

**Keywords:** Matchout deuteration, Contrast variation, SANS, Neutron scattering

## Abstract

Small-angle neutron scattering (SANS) is a powerful technique for the characterisation of macromolecular structures and interactions. Its main advantage over other solution state approaches is the ability to use D_2_O/H_2_O solvent contrast variation to selectively match out specific parts of a multi-component system. While proteins, nucleic acids, and lipids are readily distinguished in this way, it is not possible to locate different parts of a protein–protein system without the introduction of additional contrast by selective deuteration. Here, we describe new methods by which ‘matchout labelled’ proteins can be produced using *Escherichia coli* and *Pichia pastoris* expression systems in high cell-density cultures. The method is designed to produce protein that has a scattering length density that is very close to that of 100% D_2_O, providing clear contrast when used with hydrogenated partner proteins in a complex. This allows the production of a single sample system for which SANS measurements at different solvent contrasts can be used to distinguish and model the hydrogenated component, the deuterated component, and the whole complex. The approach, which has significant cost advantages, has been extensively tested for both types of expression system.

## Introduction

**Fig. 1:**
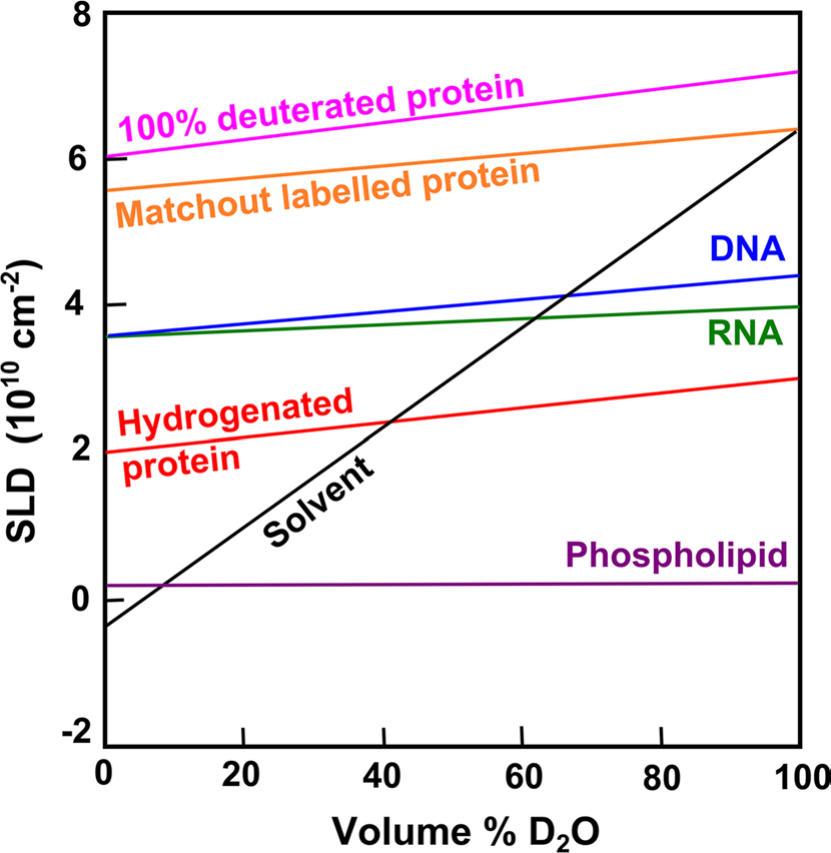
The scattering length densities (SLDs) of the four major biomolecules are depicted as a function of the volume percentage of D_2_O, assuming all labile hydrogen atoms are exchanged. The *black line* represents the variation of the solvent SLD. The match point of each biomolecule corresponds to the intersection of the solvent SLD with that for each biomolecule. Perdeuterated protein, in which all the hydrogen atoms are replaced by deuterium, has an SLD that is higher than that of D_2_O and cannot be solvent matched.

Small angle neutron scattering (SANS) and small-angle X-ray scattering (SAXS) provide important low resolution structural information on biological macromolecules in solution (Jacrot 1976; Glatter and Kratky 1982; Serdyuk et al. 2007). SANS approaches have the unique advantage of being able to exploit solvent contrast variation through the use of buffers containing specific D_2_O/H_2_O ratios (Stuhrmann 1974; Svergun et al. 2013). This capability arises from the different neutron scattering properties of hydrogen (^1^H, neutron coherent scattering length *b*_c_ = −3.7423 fm) and its heavy isotope deuterium (^2^H or D, neutron coherent scattering length *b*_c_ = 6.675 fm) (Shull 1962). This results in the very different scattering length densities (SLDs) of −0.562 × 10^10^ cm^−2^ and 6.404 × 10^10^ cm^−2^ for H_2_O and D_2_O, respectively. When specific H_2_O/D_2_O solvent mixtures are made, it is therefore possible to make solutions having any SLD in this range (Fig. 1, black line). In the normal (hydrogenated) context, the major classes of biomolecules (protein, lipid, nucleic acid) exhibit naturally occurring differences in SLD. By changing the SLD of the solvent (buffer) to match specific parts of the biomolecule being studied, each part of the complex can be individually ‘matched out’ and rendered invisible to SANS data collection (Jacrot 1976). Figure 1 shows the variation of SLD as a function of D_2_O/H_2_O composition by volume for the different classes of biomolecules, including deuterated proteins. Hydrogenated protein shows a match point at approximately 40% D_2_O, while nucleic acids show a match point of ~62%. This means, for example, that SANS data recorded for a protein-DNA complex in 40% D_2_O buffer will reveal only the DNA structure. Likewise, SANS data measured using ~62% D_2_O when the DNA is matched out will reveal the protein component alone (Fig. 1). This powerful approach enables different parts of the same complex to be modelled both separately and together (Callow et al. 2007; Niemann et al. 2008; Obarska-Kosinska et al. 2008; Rochel et al. 2011; Vijayakrishnan et al. 2011; Taylor et al. 2012; Cuypers et al. 2013; Compton et al. 2014; Appolaire et al. 2014).

**Fig. 2:**
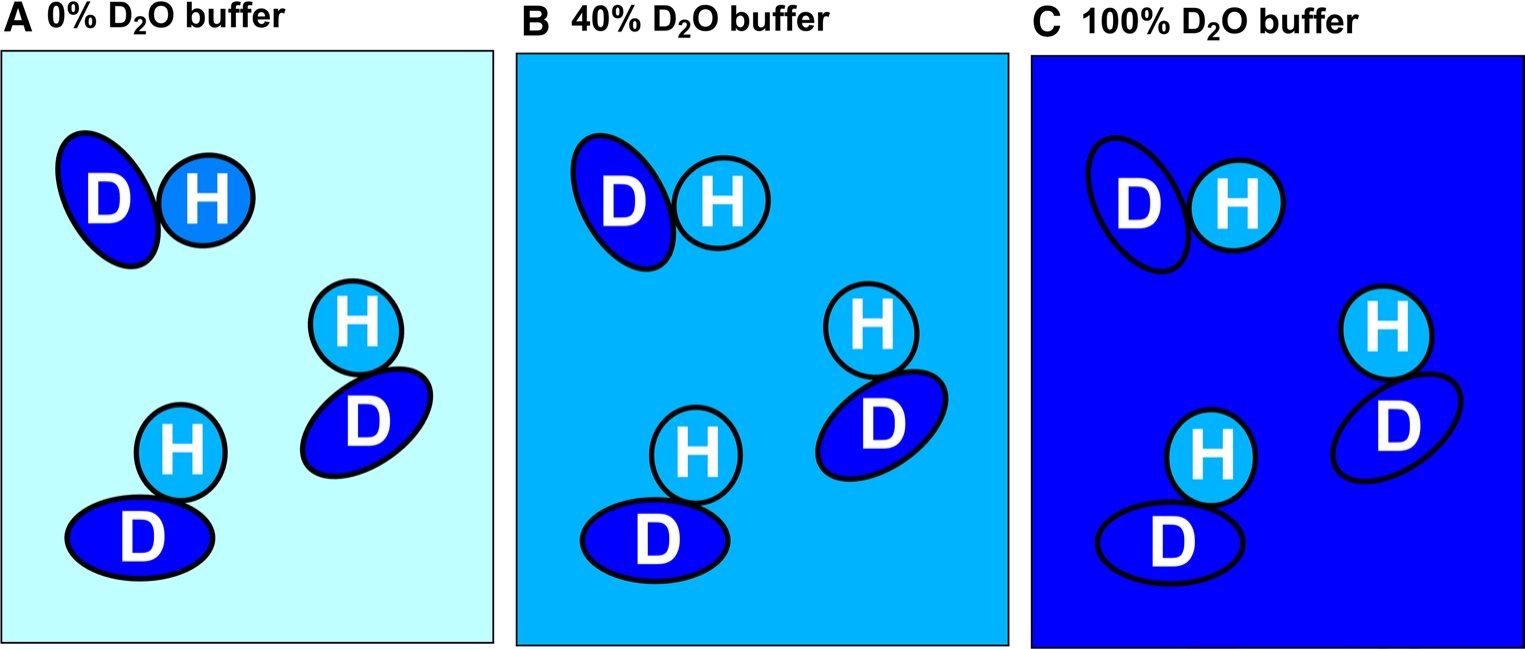
Matchout regimes for a protein–protein complex in which one protein is matchout labelled (*D*, *dark blue*) and the other is hydrogenated (*H*, *medium blue*). Following the scenarios summarised in the text, these correspond to **a** 0% D_2_O in which both protein components are visible, **b** 40% D_2_O where only the matchout labelled component (*D*) is visible, and **c** 100% D_2_O where only the hydrogenated component (*H*) is visible

To distinguish between biomolecules having the same SLD, such as protein–protein complexes, deuterium labelling is necessary. Perdeuterated protein, where all the hydrogen atoms are replaced by deuterium, has an SLD higher than that of D_2_O (Fig. 1, magenta line) and cannot be fully solvent matched in SANS experiments. However, protein that is part-deuterium labelled such that its SLD is the same as that of 100% D_2_O can be readily exploited in SANS studies. We refer to such sample material as being *matchout labelled*. This type of labelling is particularly advantageous in studies of protein–protein complexes in which one protein partner is hydrogenated and the other is matchout labelled. Here, as illustrated schematically in Fig. 2, SANS data would typically be recorded in three different buffers: (a) where the full complex is observable (e.g., 0% D_2_O), (b) where only the matchout labelled component is visible (~40% D_2_O), (c) where only the hydrogenated component is visible (100% D_2_O). In (a), there should be good correspondence between SANS and SAXS analyses of the complex, although a lower radius of gyration may be expected for the SANS analysis of the matchout-labelled protein; this will occur because it will be measured in conditions where a high fraction of the surface solvent layer around the complex will be H_2_O which has a SLD close to zero (Svergun et al. 1998; Perkins 2001).

Methods to prepare appropriately deuterated proteins for SANS are required. The *Escherichia coli* and *Pichia pastoris* expression systems are both well characterised with numerous cell lines and expression plasmids available. The effect of D_2_O in *E. coli* growth media on the deuteration of RNA polymerase and ribosomal proteins has been studied in detail (Lederer et al. 1986; Leiting et al. 1998). A method to estimate deuteration levels in whole *E. coli* cells and cellular proteins by NMR has been described (Perkins 1981). For *P. pastoris* (yeast), fewer studies have been reported, although it is clear that this yeast can grow in deuterated media (Haon et al. 1993). Here, following development work in the Deuteration Laboratory (D-Lab) of the Life Sciences group at the Institut Laue-Langevin (Haertlein et al. 2016), we describe new deuteration techniques by which matchout labelled proteins can be routinely prepared using *E. coli* and *P. pastoris*. Both expression procedures utilise cell growth in minimal media based on 85% D_2_O and a hydrogenated carbon source. The matchout labelled proteins were validated by mass spectrometry and SANS. In the case of the *E. coli* system, maltose binding protein (MBP) was used as a representative model system. MBP is a well-studied model protein that plays an important role in the metabolism of *E. coli* (Sharff et al. 1992) and is essential for the energy-dependent translocation of maltose and maltodextrins through the cytoplasmic membrane. For *P. pastoris*, a model system based on the C-terminal domain pair of human complement Factor H (CFH) was used. CFH is a key regulator of the complement system of innate immunity, in which its two C-terminal short complement regulator domains (SCR-19/20) are crucial for protecting host cells against undesired immune destruction (Rodriguez et al. 2014).

## Materials and methods

### Protein production from *E*. *coli* in matchout conditions

MBP was expressed using the *E. coli* BL21(DE3) cell line (Laux et al. 2008). High cell-density cultures were achieved using fermenters for cell growth. Minimal medium was prepared with the composition 6.86 g L^−1^ (NH_4_)_2_SO_4_, 1.56 g L^−1^ KH_2_PO_4_, 6.48 g L^−1^ Na_2_HPO_4_·2H_2_O, 0.49 g L^−1^ diammonium hydrogen citrate, 0.25 g L^−1^ MgSO_4_·7H_2_O, 1.0 mL L^−1^ (0.5 g L^−1^ CaCl_2_·2H_2_O, 16.7 g L^−1^ FeCl_3_·6H_2_O, 0.18 g L^−1^ ZnSO_4_·7H_2_O, 0.16 g L^−1^ CuSO_4_·5H_2_O, 0.15 g L^−1^ MnSO_4_·4H_2_O, 0.18 g L^−1^ CoCl_2_·6H_2_O, 20.1 g L^−1^ EDTA), and 5 g L^−1^ glycerol. The medium was supplemented with 40 mg L^−1^ kanamycin to select for the recombinant plasmid. The BL21(DE3) cells containing the DNA construct were firstly adapted to growth in minimal media using a stepwise process in which cells were inoculated into minimal media, grown for 36 h and then transferred into fresh minimal media. This was repeated until a sufficient growth rate was obtained. For the preparation of deuterated minimal media, the mineral salts were dried out in a rotary evaporator (Heidolph) at 60 °C, then dissolved in a mixture containing 85% D_2_O/15% H_2_O. Cells were then adapted to growth in deuterated media. When a sufficient growth rate was achieved, large scale expression was carried out. Then 1.5 L of deuterated medium was inoculated with 100 mL pre-culture of adapted cells in a 3 L fermenter (Labfors, Infors). During the batch and fed-batch phases, the pH was adjusted to 6.9 by addition of NaOD, and the temperature was adjusted to 30 °C. The gas-flow rate of sterile filtered air was 0.5 L min^−1^. Stirring was adjusted to ensure a dissolved oxygen tension of 30%. The fed-batch phase was initiated when the optical density at 600 nm reached 5.1. Glycerol was added to the culture to keep the growth rate stable during fermentation. When the OD_600_ reached 14.7, over-expression was induced by the addition of 1 mM IPTG and incubation continued for 24 h. Cells were then harvested and stored at −80 °C.

### Protein production from *P*. *pastoris* in matchout conditions

SCR-19/20 was expressed in yeast *P*. *pastoris* using the X-33 cell line. Cloning and expression of hydrogenated SCR-19/20 were described in detail elsewhere (Cheng et al. 2005). Cells, which had previously been transformed with the plasmid construct, were adapted to growth in a minimal media. Minimal media consisted of 13.4% yeast nitrogen base, 0.02% biotin, 1% glycerol and 100 mM potassium phosphate, pH 6.0. Adaptation was carried out by a similar protocol to that of the *E. coli* system, with the cells grown for 48 h before transferral into fresh minimal media. When high-density cell growth was achieved, cells were then adapted to growth in 85% deuterated minimal media by the same process. Deuterated minimal media was prepared as for the hydrogenated media, but was dissolved in 85% D_2_O. Large scale expression was then carried out by sustaining cell growth for 72 h in deuterated minimal media containing glycerol. Cells were then harvested by centrifugation and re-suspended in deuterated minimal media with 0.5% methanol in place of glycerol to induce protein expression. Expression was sustained for 96 h by feeding with 0.5% methanol every 24 h. All cell growth and expression was carried out using baffled flasks with shaking at 220 rpm at 29 °C. Final cell cultures were centrifuged to remove the cells from the supernatant containing the secreted protein.

### Protein purification

MBP from *E. coli* was expressed in a soluble form. Cells were broken by sonication and the insoluble fraction removed by centrifugation. For both proteins, purification was carried out using hydrogenated buffers according to the same protocol used for the hydrogenated protein. In the case of the MBP purification immobilized metal ion affinity chromatography (IMAC) on TALON (Clontech) was used. The supernatant was loaded on a column filled with 10 mL of TALON beads. This column was washed with 20 column volumes of lysis buffer containing 5 mM imidazole in 10 mM Tris–HCl, 100 mM NaCl, pH 7.5. MBP was then eluted with 100 mM imidazole. Fractions were analysed by polyacrylamide gel electrophoresis (PAGE), pooled and dialysed against 10 mM Tris–HCl, 100 mM NaCl, pH 7.5. 300 mg of MBP was obtained from 1 L of media. For the SCR-19/20 purification, cation exchange chromatography was used on an SP FF column (GE Healthcare). The supernatant was dialysed against 50 mM Tris, 25 mM NaCl, 1 mM EDTA, pH 7.4 and loaded onto the column. The column was washed with five column volumes of the same buffer. Elution was achieved by applying a NaCl salt gradient from 25 mM NaCl to 1 M NaCl. Fractions were analysed by PAGE and were pooled and dialysed against 10 mM Hepes, 137 mM NaCl, pH 7.4. Approximately 7 mg of SCR-19/20 was obtained from 1 L of start media. Further details for the MBP and SCR-19/20 purifications are given in Laux et al. (2008) and Dunne (2015) respectively.

### Mass spectrometry

Matrix-assisted laser desorption/ionization-time of flight (MALDI-TOF) mass spectroscopy was carried out on the deuterated MBP and SCR-19/20 proteins. The matrix consisted of sinapinic acid in acetonitrile/water-0.1% TFA (50:50). For both proteins, measurements were carried out at a concentration of 0.5 mg/mL in hydrogenated buffers. This meant that the calculation of the deuteration levels for the two matchout labelled proteins made the assumption that all of the labile deuterium atoms were replaced by hydrogen.

### SANS data collection

Data were collected on the SANS instruments D22 (MBP) and D33 (SCR-19/20) at the Institut Laue-Langevin, Grenoble, France (Dewhurst et al. 2016). To calculate the match point, samples were prepared in a range of D_2_O concentration in the appropriate buffer. SANS data from MBP were collected in 0, 20, 40, 60, 80% D_2_O buffers; SANS data for SCR-19/20 were collected in 0, 25, 40, 75 and 100% D_2_O buffers. Data reduction, buffer subtraction, and transmission calculations were carried out using the program GRASP. The curves were fitted using Guinier plots (Guinier and Fournet 1955) yielding the radius of gyration *R*_G_ and the scattering intensity at zero angle *I*(0). From this the normalised scattering amplitude of the proteins at each D_2_O concentration was calculated using the following expression:

$$\sqrt {\frac{I(0)}{Tcl}} ,$$

where *T* is the sample transmission, *l* is the cuvette path length (cm) and *c* is the concentration (mg/mL). This scattering amplitude was plotted as a function of D_2_O percentage and a linear fit carried out. The contrast match point was taken as the intersection of this plot on the abscissa.

## Results

*Escherichia coli* and *Pichia pastoris* cultures were successfully adapted to growth in deuterated minimal media. For both organisms, five changes of media were sufficient for adaptation. High cell densities were achieved using 3 L fermenters in which the conditions for growth were tightly controlled. For the production of *E. coli* cells in high cell-density cultures, the growth conditions were controlled and monitored using IRIS software (http://www.infors-ht.com). The induction of expression commenced at an OD of about 15. Both proteins were expressed in their soluble forms and successfully purified using a similar protocol to that used for the same hydrogenated counterparts.

### Maltose binding protein (MBP)

**Fig. 3:**
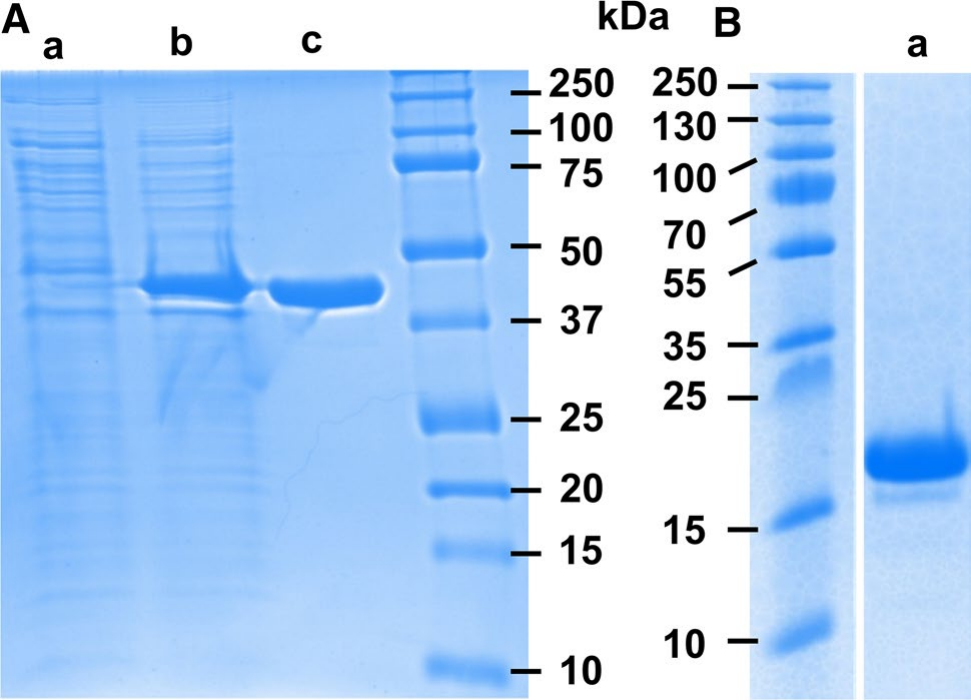
SDS-PAGE results for the expression and purification of matchout labelled protein. **A** Maltose binding protein (MBP). *a* Uninduced cellular extract; *b* extract from induced cells; *c* purified MBP after immobilized metal ion affinity chromatography. **B** SCR-19/20 from CFH. *a* After cation exchange and size exclusion chromatography

**Fig. 4:**
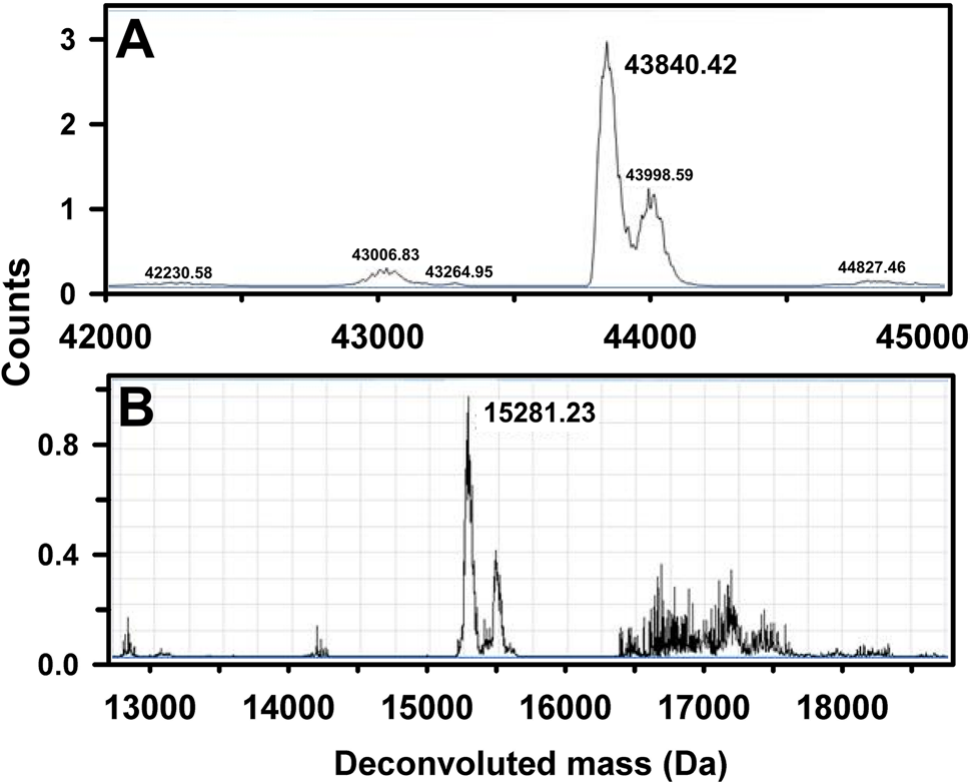
Mass spectrometry measurement (MALDI-TOF) on the two matchout-labelled proteins. **a** For MBP, the single largest peak corresponds to a molecular weight of 43,840 Da, compared with 42,360 Da for the hydrogenated analogue. **b** For SCR-19/20, the *central peak* is associated with the properly processed SCR-19/20 protein (15,281 Da)

**Fig. 5:**
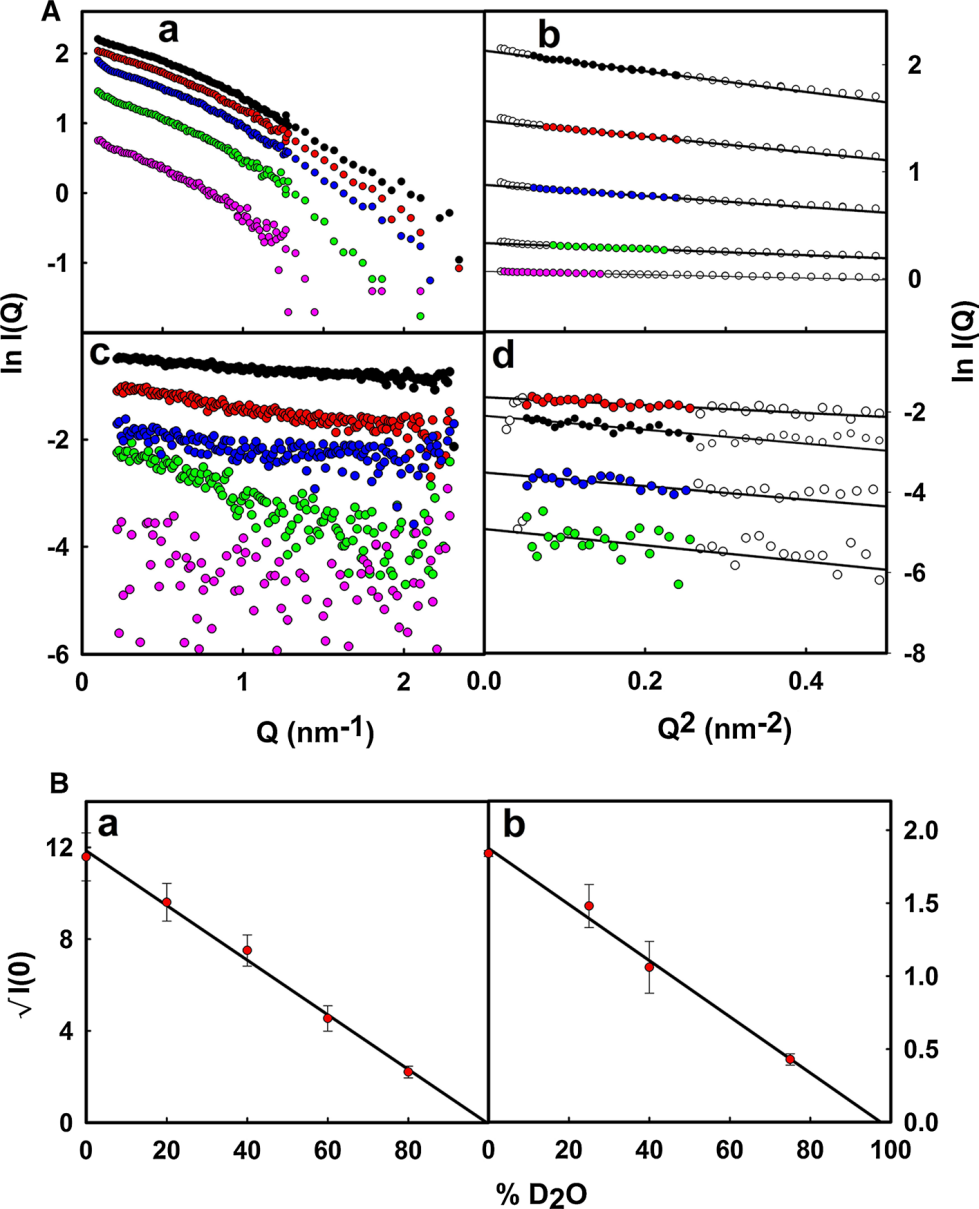
**A** *a Scattering curves* for matchout-labelled MBP in buffers containing 0% D_2_O (*black*), 20% D_2_O (*red*), 40% D_2_O (*blue*), 60% D_2_O (*green*) and 80% D_2_O (*pink*). *b* The corresponding Guinier plots for matchout-labelled MBP, with the *coloured points* representing the region of the *scattering curve* used to determine each *R*_G_ value. *c Scattering curves* for matchout-labelled SCR-19/20 in buffers containing 0% D_2_O (*black*), 25% D_2_O (*red*), 40% D_2_O (*blue*), and 75% D_2_O (*green*). *d* The corresponding Guinier plots for matchout-labelled SCR-19/20, with the *coloured points* representing the region of the *scattering curve* used to determine each *R*_G_ value. **B** *a* The analysis of $\sqrt {I(0)}$ as a function of the percentage D_2_O content in the buffer for matchout-labelled MBP expressed in *E. coli*. The matchpoint was 99.5% D_2_O. *b* The analysis of $\sqrt {I(0)}$ for matchout-labelled SCR-19/20 expressed in *P. pastoris* as a function of the percentage D_2_O content in the buffer. The matchpoint was 97% D_2_O

SDS-PAGE results before and after purification showed that MBP of high purity was obtained after a single IMAC purification step (Fig. 3A). The mass of normal hydrogenated MBP was measured to be 42,360 Da by mass spectrometry, and is identical to the value predicted from the sequence taking account for methionine aminopeptidase processing (Laux et al. 2008). Mass spectrometry measurements of the partially deuterated analogue (Fig. 4a) showed an increase in mass to 43,840 Da when measured in H_2_O buffer. The fully deuterated (aminopeptidase processed) MBP (in D_2_O solvent) was predicted to have a mass of 45,312 Da with the aid of the program ProtParam (Gasteiger et al. 2005); the deuteration level of the MBP was estimated to be 64.1% of the non-exchangeable hydrogen atoms. The smaller peak to the right of this in Fig. 4a is attributed to incomplete aminopeptidase processing of the matchout labelled MBP. The SANS data collected from MBP showed no evidence of aggregation at any contrast, and Guinier analyses were used to determine the *I*(0) and *R*_G_ values for each contrast (Fig. 5A). The *R*_G_ value of MBP was 2.6 nm. The variation of scattering amplitude $\sqrt {I(0)}$ as a function of solvent D_2_O composition for the partially deuterated MBP gave the contrast match point (Fig. 5B). That for the partially deuterated MBP expressed in a high cell-density culture with 85% D_2_O as the only source of deuterium was determined to be 99.5% D_2_O.

### Complement factor H SCR-19/20

Deuterated SCR-19/20 of high purity was obtained by cation exchange chromatography followed by size exclusion chromatography. This was confirmed by the PAGE results after purification (Fig. 3B). The yield of deuterated SCR-19/20 was calculated from the absorbance (A280 nm) of the pooled size exclusion chromatography fractions corresponding to the protein peak. From 1 L of glycerol growth media, an average of 7.2 mg protein was obtained. This yield was similar to the average of 7 mg obtained for hydrogenated SCR-19/20 in hydrogenated nutrient rich media. The mass of hydrogenated SCR-19/20 was 14,734 Da. Mass spectrometry showed that the mass increased to 15,281 Da following deuteration (Fig. 4b). Because perdeuterated SCR-19/20 has an expected mass of 15,749 Da, the deuteration level was deduced to be 71.4% of the non-exchangeable hydrogen atoms. An additional peak was observed at 15,490 Da, corresponding to two additional amino acids, and was attributed to erroneous Kex protease processing of the signal peptide.

The SANS data for SCR-19/20 also showed no evidence of protein aggregation, and resulted in the *I*(0) and *R*_G_ values for each contrast in Guinier plots (Fig. 5A). The *R*_G_ value was 2.2 nm for SCR-19/20. The scattering amplitude plot of $\sqrt {I(0)}$ for the partially deuterated SCR-19/20 gave a contrast match point of 97% D_2_O and confirms that our protocol was optimal for the production of matchout labelled protein (Fig. 5B).

## Discussion and conclusion

The novel deuteration methods described here allow the efficient production of matchout labelled proteins that are optimised for SANS structural studies in solution. For both proteins studied, the yields were similar to those observed for hydrogenated nutrient rich media. The difference in the deuteration levels (for the non-exchangeable hydrogen atoms) of the two proteins, as observed by mass spectrometry, are attributed to their different amino acid compositions and to differences in the growth media used. In both cases a ~100 Da broadening of the main peak was observed by mass spectrometry, compared to the hydrogenated or perdeuterated proteins, and this reflects the nature of the random fractional deuteration regime used. For a typical SANS experiment, this labelling heterogeneity is not normally a problem. Proteins that are matchout labelled in this way are particularly useful in structural analyses of protein–protein complexes for which one protein of the pair would be labelled.

The advantages of this method of labelling are:

1. Matchout labelled proteins are very effective in SANS studies of complexes. Only a single preparation of complex containing a matchout labelled component is required to allow measurements for a complete SANS structural analysis. Measurements at 100%, ~40%, and 0% D_2_O solvent contrasts will yield structural information on the unlabelled component, the labelled component, and the full complex respectively, i.e., on conformational changes in either protein after complex formation, as well as the relative orientation of the two proteins in the complex. Such a study requires that the complex is stable (i.e., a low dissociation constant) and that there is no propensity for aggregation in all the required solvents. The approach has significant advantages over SANS experiments in which two separately labelled complexes are produced in which one of the two components is typically perdeuterated, and has already been successfully applied to numerous protein complexes.
2. The adaptation of host cells to growth conditions containing a hydrogenated carbon source and 85% D_2_O is much more efficient than the culture conditions needed for perdeuteration.
3. Significant cost advantages are accrued, because no deuterated carbon source is needed in the culture medium. Since the solvent used is ~85% D_2_O, this permits the effective use of recycled D_2_O. Previously-described methods used more expensive deuterated glycerol and deuterated methanol to obtain similar yields of protein (Massou et al. 1999; Pickford and O’Leary 2004).

The ability to label proteins using either *E. coli* or *P. pastoris* expression systems offers versatility in the labelling of recombinant proteins. For those systems where there are difficulties in expressing folded protein in *E. coli*, the use of *P. pastoris* offers a robust alternative in which issues associated with co-translational and post-translational modification are addressed. Future work of this type will focus on the development of similar matchout labelling approaches for insect cells and mammalian cells.
